# Quality of life and mental health in children and adolescents during the first year of the COVID-19 pandemic: results of a two-wave nationwide population-based study

**DOI:** 10.1007/s00787-021-01889-1

**Published:** 2021-10-12

**Authors:** Ulrike Ravens-Sieberer, Anne Kaman, Michael Erhart, Christiane Otto, Janine Devine, Constanze Löffler, Klaus Hurrelmann, Monika Bullinger, Claus Barkmann, Nico A. Siegel, Anja M. Simon, Lothar H. Wieler, Robert Schlack, Heike Hölling

**Affiliations:** 1 Department of Child and Adolescent Psychiatry, Psychotherapy, and Psychosomatics, University Medical Center Hamburg-Eppendorf, Hamburg, Germany; 2 Alice Salomon University of Applied Sciences, Berlin, Germany; 3 Apollon University of Applied Sciences, Bremen, Germany; 4 Argora Clinic, Psychosomatic Clinic and Outpatient Center, Berlin, Germany; 5 Hertie School, Berlin, Germany; 6 Department of Medical Psychology, University Medical Center Hamburg-Eppendorf, Hamburg, Germany; 7 Infratest Dimap, Berlin, Germany; 8 Robert Koch Institute, Berlin, Germany; 9 Department of Epidemiology and Health Monitoring, Robert Koch Institute, Berlin, Germany

**Keywords:** COVID-19, Mental health, Quality of life, Anxiety, Depression, Children and adolescents

## Abstract

**Background:**

The COVID-19 pandemic has disrupted the lives of children and adolescents worldwide. The German COPSY study is among the first population-based longitudinal studies to examine the mental health impact of the pandemic. The objective of the study was to assess changes in health-related quality of life (HRQoL) and mental health in children and adolescents and to identify the associated risk and resource factors during the pandemic.

**Methods:**

A nationwide longitudinal survey was conducted with two waves during the pandemic (May/June 2020 and December 2020/January 2021). In total, *n* = 1923 children and adolescents aged 7 to 17 years and their parents participated (retention rate from wave 1 to wave 2: 85%). The self-report and parent-proxy surveys assessed HRQoL (KIDSCREEN-10), mental health problems (SDQ with the subscales emotional problems, conduct problems, hyperactivity, and peer problems), anxiety (SCARED), depressive symptoms (CES-DC, PHQ-2) and psychosomatic complaints (HBSC-SCL). Mixed model panel regression analyses were conducted to examine longitudinal changes in mental health and to identify risk and resource factors.

**Results:**

The HRQoL of children and adolescents decreased during the pandemic, and emotional problems, peer-related mental health problems, anxiety, depressive and psychosomatic symptoms increased over time, however the change in global mental health problems from wave 1 to wave 2 was not significant, and some changes were negligible. Socially disadvantaged children and children of mentally burdened parents were at particular risk of impaired mental health, while female gender and older age were associated with fewer mental health problems. A positive family climate and social support supported the mental health of children and adolescents during the pandemic.

**Discussion:**

Health promotion, prevention and intervention strategies could support children and adolescents in coping with the pandemic and protect and maintain their mental health.

**Supplementary Information:**

The online version contains supplementary material available at 10.1007/s00787-021-01889-1.

## Introduction

The coronavirus disease 2019 (COVID-19) pandemic has significantly changed the lives of billions of people worldwide. Confronted with currently more than four million deaths and more than 200 million cases, most countries have implemented extensive public health infection prevention measures. Children and adolescents have to cope with social distancing rules, temporary school closures and massive restrictions on their leisure activities. Experts have raised concerns that these measures have tremendous impacts on the mental health of children and adolescents [1].

There is increasing empirical evidence of psychological distress among children and adolescents during the pandemic according to systematic reviews of mostly cross-sectional studies [2, 3]. These reviews describe an average doubling of clinically elevated anxiety (21%) and depression (25%) symptoms [2] and high pooled prevalences of depression (29%), anxiety (26%), sleep disorders (44%), and posttraumatic stress symptoms (48%), respectively [3]. International cross-sectional studies further report lower levels of health-related quality of life (HRQoL) of children and adolescents comparing data from a population-based study gathered before the pandemic with data of another sample gathered during the pandemic [4]. Relationships with friends are impaired, homeschooling is perceived as exhausting, and family conflicts tend to increase [4, 5]. Pediatric experts warn that the risks of child abuse and neglect are increasing during the pandemic [6]. Socially disadvantaged children and children with pre-existing mental health problems appear to be particularly at risk of being adversely affected [4, 7]. There are concerns that mental health problems may remain undiagnosed and untreated due to the closure of schools and treatment centers [5].

Researchers and politicians are calling for longitudinal studies to determine the long-term consequences of the pandemic and to explore the risks and resources involved in mental health trajectories [8]. To date, there have been very few longitudinal studies, and most of those that exist compared mental health outcomes before and during the pandemic. Those studies indicate that mental health problems in children and adolescents have increased [9, 10]. Large population-based longitudinal studies monitoring changes during the pandemic are lacking. The British Co-SPACE study is the first to report longitudinal results on child mental health during the pandemic. The authors found large increases in hyperactivity/inattention with a standardized mean difference (SMD) of 0.22, and small increases in conduct problems (SMD: 0.16) and emotional symptoms (SMD: 0.05) during the first lockdown in children aged 4–10 years in the UK (March to May 2020); in adolescents (11–16 years), the authors found an increase which was only minimal in hyperactivity/inattention (SMD: 0.04), little in conduct problems (SMD: 0.02), and a slight decrease of emotional problems over time (SMD: − 0.09) [10]. Another longitudinal study among adolescents and young adults aged on average 17 years in New York found that symptoms of anxiety and depression initially increased during the early months of the pandemic, when infection rates peaked (March–April 2020), and then decreased during the summer months with home confinement and school closures in place and infection rates going down (May–July 2020) [11]. Further longitudinal studies monitoring child and adolescent mental health are currently being conducted in Australia, Canada and Scotland, but their results have not yet been published.

The German COPSY study (Impact of COVID-19 on Psychological Health) is among the first population-based longitudinal studies to monitor HRQoL and mental health in children and adolescents aged 7–17 years at two time points during the pandemic. Furthermore, population-based data from the Behaviour and Wellbeing of Children and Adolescents in Germany (BELLA) study [12] and the international Health Behaviour in School-aged Children (HBSC) study [13] were used for comparisons with pre-pandemic data.

The study aims to address the following research questions:

1. How did the HRQoL and mental health of children and adolescents change from wave 1 to wave 2 of the COPSY study?
2. Which risk and resource factors are associated with (a) HRQoL and mental health in children and adolescents during the pandemic and (b) with changes in HRQoL and mental health across wave 1 and wave 2?

Based on conceptual considerations and previous studies, we expected a sizeable decrease in HRQoL and a strong increase in mental health problems and psychosomatic complaints compared to pre-pandemic data. We also expected a slight worsening in these outcomes from wave 1 to wave 2. It was also hypothesized that the well-known determinants of HRQoL and mental health (e.g., low socio-economic status, parental mental illness, family conflicts) are risk factors for lower HRQoL and more pronounced mental health problems and psychosomatic complaints. On the other hand, positive family climate and social support were assumed to serve as resources associated with higher HRQoL and fewer mental health problems. Please note that the COPSY study does not employ psychiatric diagnoses but uses well-established, standardized, and validated screening instruments to assess HRQoL and the risk for mental health problems. However, in line with accepted designations for what is measured by these instruments, the notation of HRQoL and mental health problems will be used in this paper.

## Methods

### Study design and sample

**Fig. 1:**
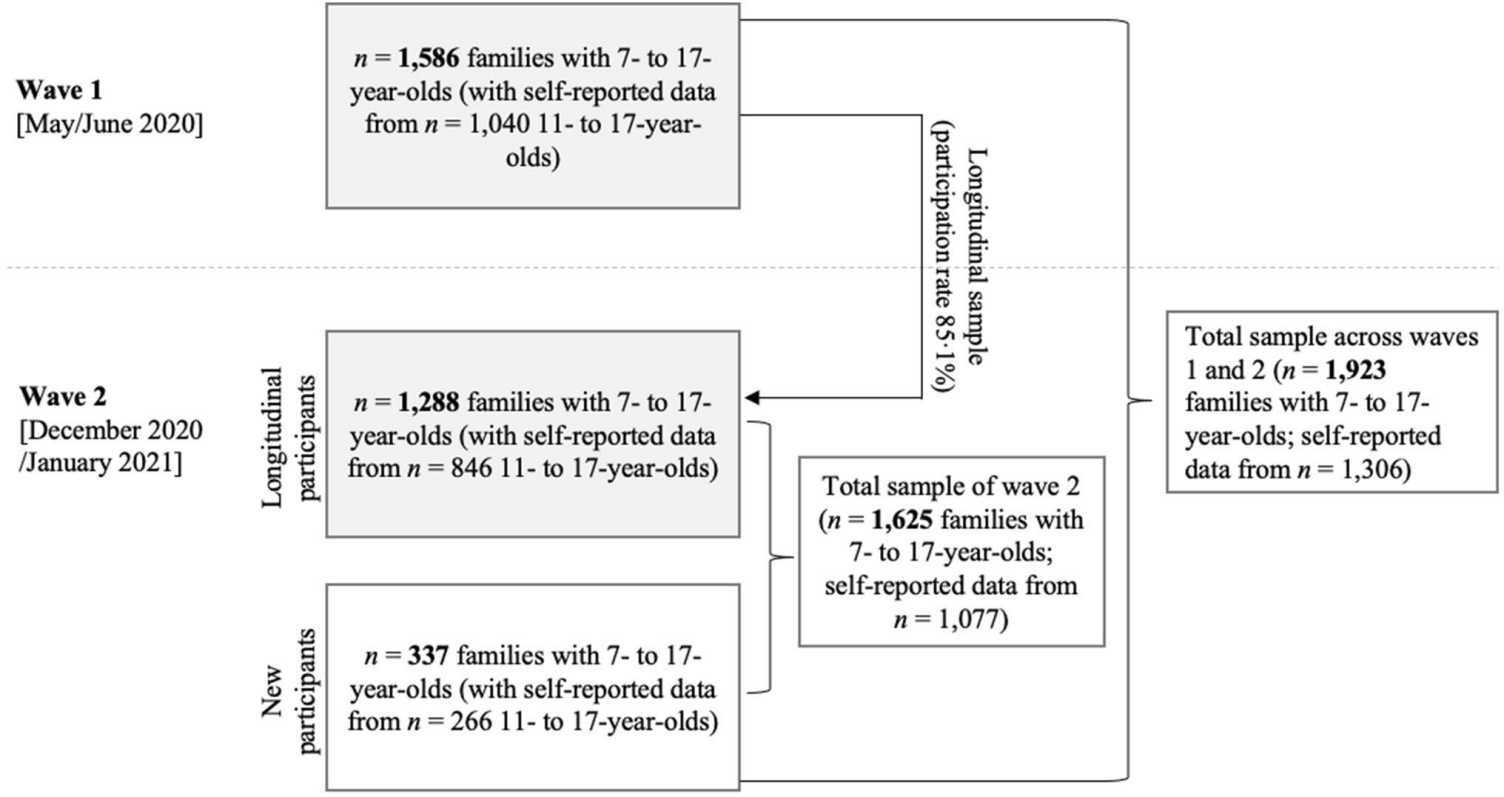
Numbers of participating families, children and adolescents in wave 1 and wave 2 of the COPSY study

The first wave of the population-based longitudinal COPSY study was conducted from May 26 to June 10, 2020, shortly after the peak of the first wave of the COVID-19 pandemic (03/04/2020) in Germany. Daycares, schools, cultural institutions and stores (other than grocery stores) had been completely locked down for about 8 to 10 weeks and started to slowly reopen again (early May to mid-June 2020). So all German children and adolescents had been deprived of peer contact (except if they had parents working in systemically relevant jobs which allowed the children to go to emergency childcare) and contacts with their grandparents as well as participation in most leisure/sports activities for at least 2 months. The population-based study design of the COPSY study and first results have been described elsewhere [4]. We collected nationwide self-reported data from children and adolescents aged 11–17 years (*n* = 1040) and parent-reported data for 7- to 17-year-olds (*n* = 1586). The second wave of the study was conducted between December 17, 2020, and January 25, 2021, during the second wave of the pandemic (10/2020 to 01/2021), which led to daycare and school closures as well as a complete lockdown of all stores (except grocery stores) and all cultural/leisure institutions between mid-December 2020 and the 10th of January 2021. After January 10th those measures were slowly loosened again, with the speed of relaxing the measures varying across German regions. In our study, we used short periods for data assessments since we wanted to avoid potential effects of changes in measures of infection control as implemented by the government during the pandemic. Families were recruited through a population-based approach from an online panel using quota sampling to ensure that the recruited sample matched with sociodemographic characteristics of the overall German population (according to the Microcensus 2018; considering gender, age, education, and region; 50 cells were created based on the characteristics and cross quotation was further used). Families who had already participated in wave 1 (*n* = 1586) were approached online and via mail, informed about the study and asked for their informed consent before participating in wave 2. With a participation rate of 85.1%, *n* = 1288 families re-participated in wave 2 and completed the online survey. All participants were recruited from all over Germany and received a small incentive (e.g., children got a postcard and could choose between different keychains). Using additional quota sampling, *n* = 337 new families were recruited to compensate for drop-outs from waves 1 to 2 in order to guarantee sociodemographic representativeness and comparability across waves 1 and 2. Additional sampling was needed as further follow-up surveys of the COPSY study are planned. Thus, the additional sampling was necessary to account for drop out and is an effort to keep the retention rate as high as possible. Overall, self-reported data were collected from 11- to 17-year-olds (*n* = 1077), and parent-reported data were collected for 7- to 17-year-olds (*n* = 1625) in wave 2. The overall sample size across both waves was *n* = 1923 families with self-reported data from *n* = 1306 children and adolescents (see Fig. 1).

In a responder versus non-responder analysis, we compared families who participated in both waves (*n* = 1288) with those who only participated in wave 1 but not in wave 2 (*n* = 298). No significant differences were found in sociodemographic or mental health-related variables. The data sets from waves 1 and 2 were each weighted to correspond to the sociodemographic characteristics of the German population (according to the 2018 Microcensus; the individual weights ranged from 0.2 to 3.8 for wave 1 and from 0.2 to 4.0 for wave 2). The COPSY study was approved by the Local Psychological Ethics Committee (LPEK-0151) and the Commissioner for Data Protection of the University of Hamburg.

### Measures

#### Sociodemographic variables

The age and gender of the children and adolescents and their parents were assessed. The parents also reported their marital status, occupation, education, living space, and migration background. Parental education was assessed by two items asking for the highest academic and vocational qualification of both parents. According to the international “Comparative Analysis of Social Mobility in Industrial Nations” (CASMIN) classification, a categorization into parents with low, medium and high education was performed [14].

#### COVID-19 burden

Children and adolescents as well as their parents were informed about the study aim to investigate the impact and consequences of the pandemic on the psychosocial health of children and adolescents/families and were asked about the perceived burden of the pandemic, both overall and that specifically caused by social distancing, school closures, changes in family atmosphere and changes in occupational status. The items were newly developed and can be found in Supplementary Table 1; due to the novelty of the pandemic and the need for timely assessment, these items have not been validated psychometrically before. However, they have been pilot tested (including cognitive interviews) with 50 children and their parents prior to the main assessment for feasibility, comprehensibility, and length.

#### HRQoL and mental health

We followed the recommendations of the International Consortium for Health Outcomes Measurement [15] with regard to administering established, standardized, and validated questionnaires. HRQoL in children and adolescents was measured using the self-report version of the 10-item KIDSCREEN-10 Index [16], which has good internal consistency (Cronbach’s *α* = 0.82), good retest reliability (ICC = 0.70) and good validity as shown in several studies [17–19]. Children and adolescents with low, normal or high HRQoL were differentiated based on reference scores from the BELLA study (normal HRQoL was defined as M_BELLA_ ± 1SD_BELLA_). The Strengths and Difficulties Questionnaire (SDQ) [20] was administered to assess the children’s mental health problems as reported by their parents. It is a very established and widely used measure with satisfactory reliability (Cronbach’s *α* = 0.73), cross-informant correlation (mean: 0.34), retest reliability (0.62) and validity [21]. The SDQ provides a total difficulties score across 20 items and four subscales regarding emotional problems, conduct problems, hyperactivity, and peer problems. Published cut-off values were used to discriminate participants with and without mental health problems (noticeable/abnormal and borderline vs. normal) [22]. Self-reported anxiety in children and adolescents was measured using the 9-item general anxiety subscale of the Screen for Child Anxiety Related Disorders (SCARED) [23], an instrument, which has shown good internal consistency (Cronbach’s *α* = 0.74–0.93), good test–retest reliability (ICC = 0.70–0.90) and good validity in several studies [23]. Based on published cut-off values [23], groups of participants with and without anxiety were created. Self-reported depressive symptoms were assessed with seven items of the German version of the Center for Epidemiological Studies Depression Scale (CES-DC) [24], which has shown good internal consistency (Cronbach’s *α* = 0.84–0.87). A mean score gathering all items was calculated with higher scores indicating more severe depressive symptoms. In addition, the prevalence of depressive symptoms was assessed using the 2-item Patient Health Questionnaire (PHQ-2) [25], an internationally widely used, established depression screening instrument with good internal consistency (Cronbach’s *α* = 0.83) reliability (*r* = 0.67–0.87) and validity [26]. A published cut-off value was used to categorize participants into those with and without noticeable depressive symptoms [25]. The KIDSCREEN-10 Index, the SDQ and the SCARED were administered in the BELLA study as well, allowing comparisons across both studies. The HBSC symptom checklist (HBSC-SCL) [27] was adopted to measure the frequencies of self-reported psychosomatic complaints during the past week. The HBSC-SCL has shown acceptable unidimensionality, internal consistency, and test–retest reliability (*r* = 0.61–0.79) as well as international comparability [28]. For each psychosomatic symptom, we differentiated participants into groups of those who had experienced the respective symptom at least once peer week vs. those who had experienced it less frequently. The mean item score was calculated as an overall measure, with higher values indicating more complaints. The HBSC-SCL allows comparisons to be made between the COPSY and HBSC studies.

#### Psychosocial risk and resource factors

Parents provided information on any current psychiatric conditions that they themselves had been diagnosed with by a psychologist or physician. Familial resources were assessed using four items from the self-reported Family Climate Scale [29]; in the BELLA study an 8-item version of the measure was used and showed acceptable-to-good internal consistency (Cronbach’s *α* = 0.78–0.83) [30]. Social support was measured using four items from the Social Support Scale [31], an objective, reliable and valid instrument (e.g., Cronbach’s *α* = 0.97) [32, 33].

### Data analysis

First, absolute and relative frequencies of the categorical HRQoL and mental health outcomes were calculated based on weighted data from wave 2 and then compared with data from wave 1 and with pre-pandemic data from the BELLA and HBSC studies. To test longitudinal changes in HRQoL and mental health scores from wave 1 to wave 2 and to identify risk and resource factors associated with HRQoL and mental health within (longitudinal) and between (cross-sectional) respondents, mixed model panel regression analyses were conducted. Coefficients were estimated representing a potential effect of the time of the pandemic and lockdown (0 = wave 1; 1 = wave 2), time-constant risk factors (i.e., age, gender, migration background, parental education, single parenthood, living space and parental mental illness), pandemic-related risk factors (i.e., parental burden caused by the pandemic and by changes in occupational status, family conflicts and the escalation of conflicts) and resource factors (i.e., family climate and social support). The simultaneous inclusion of these predictors in the multivariate models allows for thoroughly controlling for confounding factors as well. In each model, a random intercept was included for every participant (to allow and represent individually differing scores). To identify factors associated with changes in HRQoL and mental health across waves 1 and 2, interaction terms between the time of the pandemic and lockdown and the time-constant variables were included as additional predictors. The random effects panel model was chosen because it allows the simultaneous inclusion of time-constant and time-varying covariates [34].

The required sample size was calculated using G-Power 3.1 software and based on a cut-off for statistical significance of *p* (alpha) < 0.05 and a power of 80% for a small effect (*f* = 0.1) between waves 1 and 2 (within factor) and between two groups (between factor), and an interaction between waves 1 and 2 and two groups (within between interaction). We did not correct the two-sided alpha level using Bonferroni correction because the comparisons are not completely independent from each other and outcomes as well as predictors are interrelated to a certain degree. This resulted in minimum sample sizes of *n* = 200 and *n* = 592, respectively. The panel analyses were conducted using the NLME package (version 3.1–152) in R (version 4.04). For the remaining analyses, SPSS version 26 was used.

## Results

### Sociodemographics

Of *n* = 1923 families with 7- to 17-year-old children (*M*_age_ = 12.67, SD_age_ = 3.29, 49.7% female), the majority had no migration background (83.6%), more than half of the participating parents had a moderate level of education (56.1%) and about half of them were employed full-time (51.5%). In total, 18.4% were single parents. Further details on the sociodemographic characteristics of the sample are given in Table 1.

**Table 1:** Sociodemographic characteristics of the COPSY sample

	Children and adolescents aged 7–17 years (parent-report)		Children and adolescents aged 11–17 years (self-report)	
	(*n* = 1923)		(*n* = 1306)	
	*n* (%)	*M* (SD)	*n* (%)	*M* (SD)
Age		12.67 (3.29)		14.56 (2.00)
7–10 years	606 (31.5)		–	
11–13 years	392 (20.4)		385 (29.5)	
14–17 years^a^	925 (48.1)		921 (70.5)	
Gender				
Male	964 (50.1)		638 (48.9)	
Female	956 (49.7)		666 (51.9)	
Other	2 (0.1)		2 (0.2)	
Age of the parent, years		44.05 (7.35)		45.99 (6.95)
Migration background				
No	1608 (83.6)		1,100 (84.2)	
Yes	315 (16.4)		206 (15.8)	
Parental education				
Low	342 (17.8)		230 (17.6)	
Moderate	1079 (56.1)		708 (54.2)	
High	478 (24.9)		352 (27.0)	
No information	24 (1.2)		16 (1.2)	
Single parenthood				
No	1569 (81.6)		875 (80.3)	
Yes	354 (18.4)		257 (19.7)	
Occupational status				
Full-time employed	990 (51.5)		695 (53.2)	
Part-time employed	555 (28.9)		369 (28.3)	
Self-employed	78 (4.1)		58 (4.3)	
Other employment	34 (1.8)		25 (1.9)	
Housewife/househusband	130 (6.8)		76 (5.8)	
Retiree/pensioner	42 (2.2)		33 (2.5)	
On parental leave	26 (1.4)		8 (0.6)	
Unemployed	68 (3.5)		44 (3.4)	
COVID-19				
A family member contracted COVID-19	207 (10.8)		139 (10.6)	
A relative died of COVID-19	78 (4.1)		56 (4.3)	

Unweighted data

*M* mean; *SD* standard deviation

^a^*n* = 60 adolescents had already turned 18 when they participated in the second wave of the survey, but were included in the age group of 14- to 17-year-olds

### Perceived burden of the pandemic

In wave 1 of the COPSY study, more than two-thirds (69.4%) of the 11- to 17-year-old children and adolescents reported being burdened by the pandemic; the corresponding proportion was significantly higher in wave 2, the corresponding effect size indicated a small effect for this difference between waves 1 and 2 (82.6%; *p* < 0.001; *ϕ* = 0.15). In both waves, more than half of the participants stated that attending school and learning were more difficult than before the pandemic (wave 1: 64.4%, wave 2: 63.9%; *p* = 0.717). Furthermore, the majority reported fewer social contacts than before the pandemic at both waves, with a significantly higher proportion at wave 1 compared to wave 2, though the corresponding effect was negligible (wave 1: 82.8%, wave 2: 76.1%; *p* < 0.001; *ϕ* = 0.07). At both waves, about two-fifths reported impairments of their relationships with their friends (wave 1: 37.9%, wave 2: 39.4%; *p* = 0.475). Approximately one-fourth of the 11- to 17-year-olds stated that family arguments had increased compared to the time before the pandemic at both waves (wave 1: 26.2%, wave 2: 23.8%; *p* = 0.191).

### HRQoL and mental health in children and adolescents during the pandemic

**Fig. 2:**
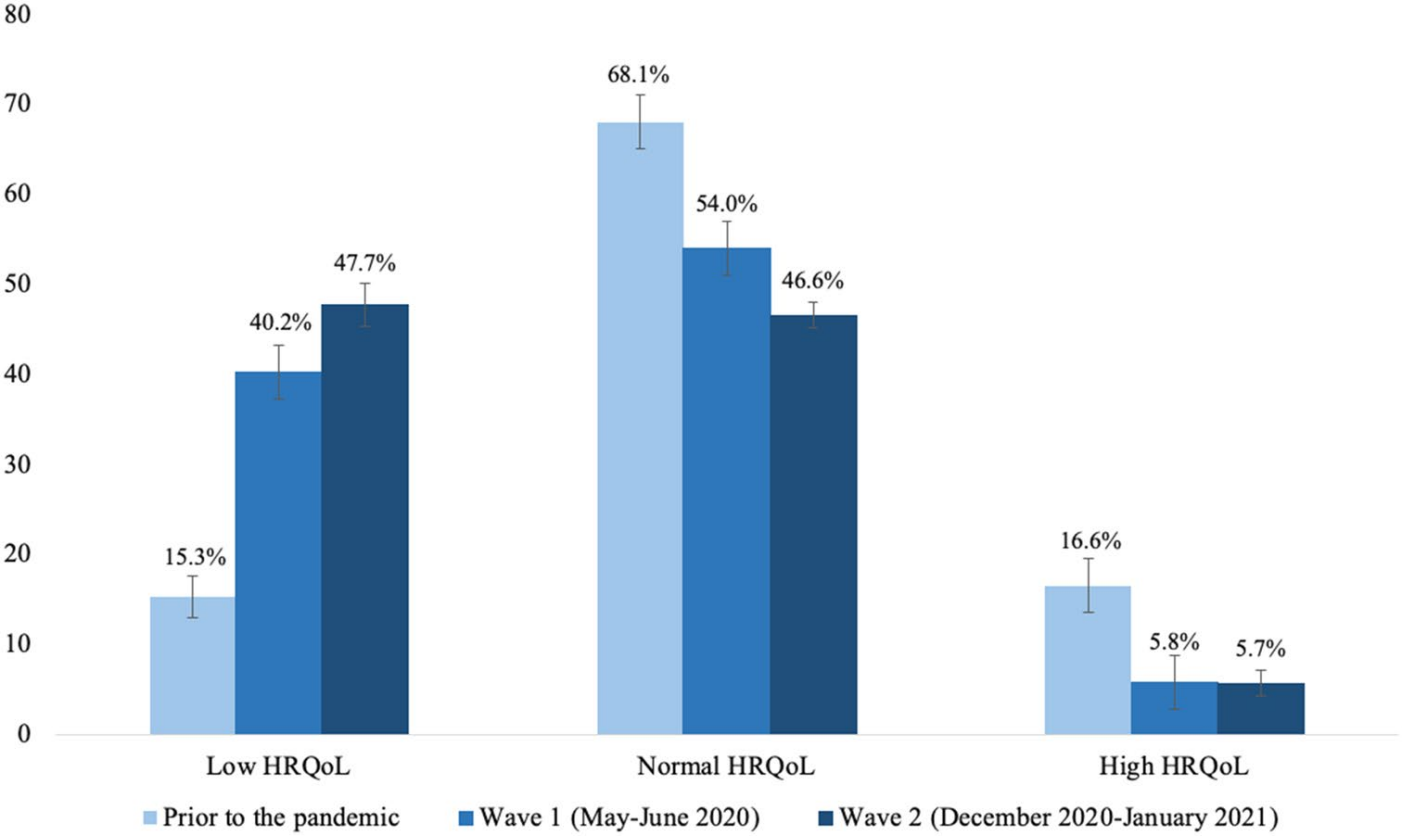
Health-related quality of life of children and adolescents measured prior to the COVID-19 pandemic (BELLA study), in wave 1 (*n* = 1586) and in wave 2 (*n* = 1625) of the COPSY study. Significant differences in HRQoL prior vs. wave 1, prior vs. wave 2 and wave 1 vs. wave 2

**Fig. 3:**
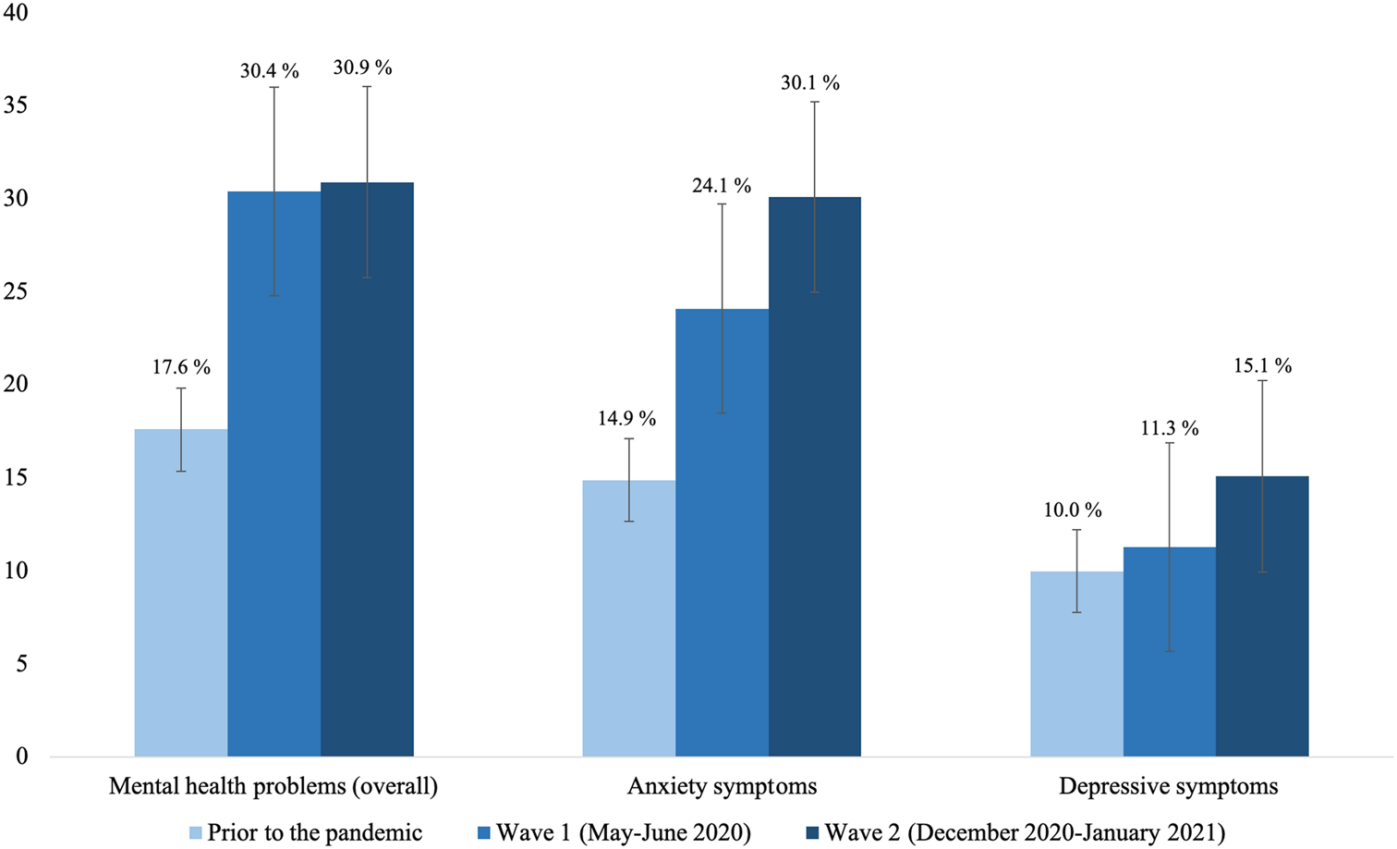
Mental health problems, anxiety symptoms and depressive symptoms measured prior to the COVID-19 pandemic (BELLA study), in wave 1 and in wave 2 of the COPSY study. Significant differences in mental health problems prior vs. wave 1 and prior vs. wave 2; significant differences in anxiety prior vs. wave 1, prior vs. wave 2 and wave 1 vs. wave 2; significant differences in depression prior vs. wave 2 and wave 1 vs. wave 2

**Fig. 4:**
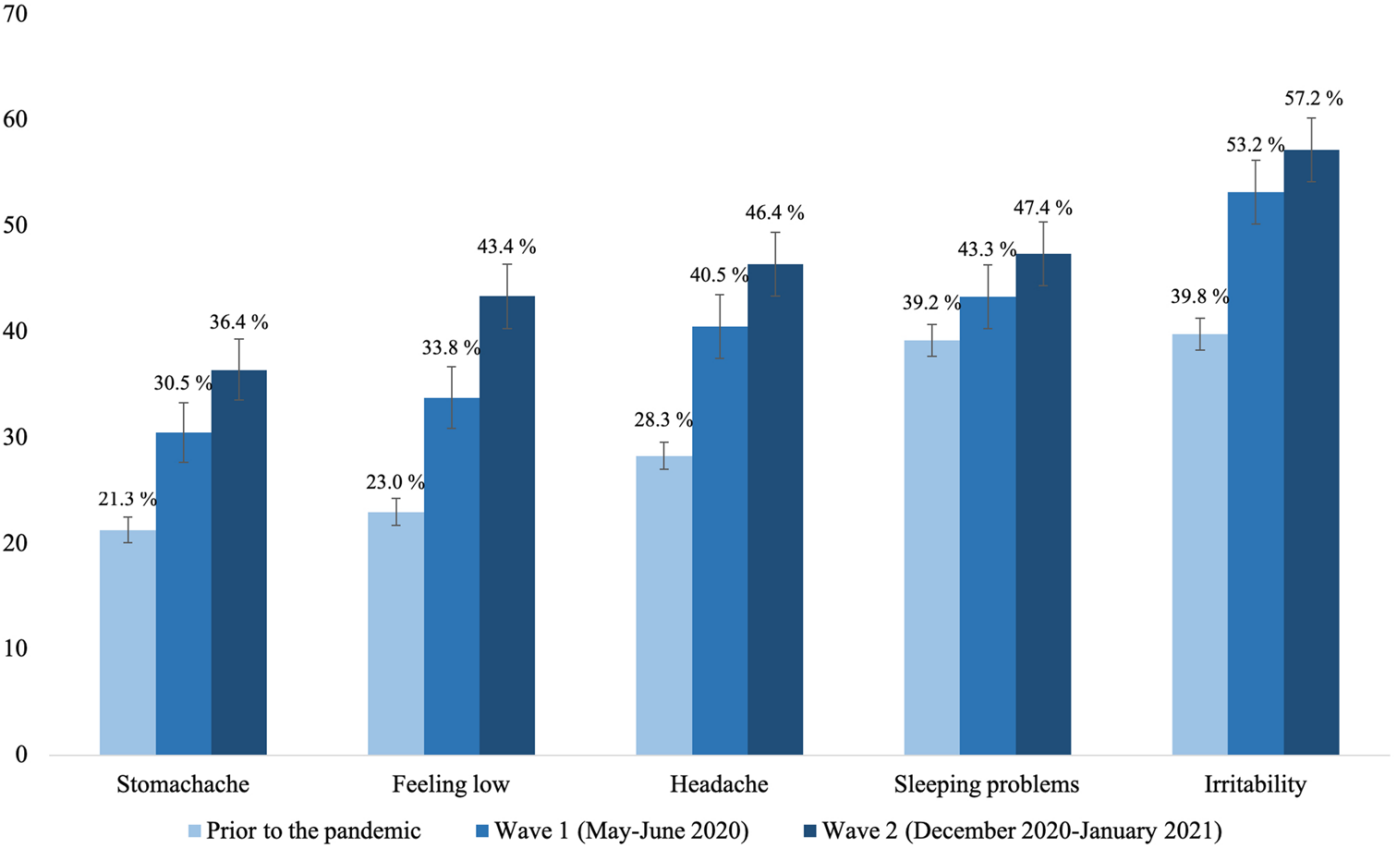
Psychosomatic complaints at least once per week measured prior to the COVID-19 pandemic (BELLA study), in wave 1 and in wave 2 of the COPSY study. Significant differences in stomachache, feeling low, and headache prior vs. wave 1, prior vs. wave 2 and wave 1 vs. wave 2; significant difference in sleeping problems and irritability prior vs. wave 1 and prior vs. wave 2

The HRQoL and mental health of children and adolescents was lower during the pandemic (wave 1 and 2) compared to pre-pandemic data. In wave 2, 47.7% of the 11- to 17-year-olds reported low HRQoL (wave 1: 40.2%, pre-pandemic: 15.3%; see Fig. 2). The difference in HRQoL was significant between pre-pandemic data and COPSY wave 1 data [4]; further, the difference between HRQoL data from COPSY waves 1 and 2 was significant as well, though the effect was negligible (*p* < 0.001; *ϕ* = 0.08). A proportion of 30.9% suffered from mental health problems, such as conduct problems, hyperactivity, peer problems and emotional problems (wave 1: 30.4%, pre-pandemic: 17.6%; see Fig. 3); the difference between pre-pandemic and wave 1 data was significant (*p* < 0.001; compare [4]), but no significant difference was found comparing waves 1 and 2 (*p* = 0.706). A proportion of 30.1% had symptoms of generalized anxiety (wave 1: 24.1%, pre-pandemic: 14.9%; see Fig. 3); the difference between pre-pandemic and wave 1 data was significant [4], the difference between waves 1 and 2 was significant as well, but negligible due to the effect size (*p* = 0.002; *ϕ* = 0.07). At wave 2 of the COPSY study, 63.0% had trouble concentrating (wave 1: 62.1%), 61.9% had little interest or joy in activities (wave 1: 58.4%), and 39.8% felt sad (wave 1: 33.7%). The observed difference between wave 1 and 2 was statistically significant only for the item “I felt sad” (*p* = 0.004). According to the PHQ-2, 15.1% had depressive symptoms (wave 1: 11.3%, pre-pandemic: 10.0%; see Fig. 3); contrary to our expectations, no significant difference in depressive symptoms was found between pre-pandemic and COPSY wave 1 data [4] and the difference between wave 1 and wave 2 data was significant, but negligible (*p* = 0.010; *ϕ* = 0.01). Furthermore, children and adolescents reported psychosomatic complaints such as irritability (wave 2: 57.2%, wave 1: 53.2%, pre-pandemic: 39.8%), headaches (wave 2: 46.4%, wave 1: 40.5%, pre-pandemic: 28.3%), stomachaches (wave 2: 36.4%, wave 1: 30.5%, pre-pandemic: 21.3%), and feeling low (wave 2: 43.4%, wave 1: 33.8%, pre-pandemic: 23.0%; see Fig. 4). Differences between pre-pandemic and COPSY wave 1 data have already been reported [35]. Comparing wave 1 and wave 2 data, significant differences indicated higher proportions of children affected by headaches (*p* = 0.007), stomachaches (*p* = 0.004) and feeling low (*p* < 0.001); the corresponding effect sizes were negligible for headaches and stomachaches (*ϕ* = 0.06 for both), and small for feeling low (*ϕ* = 0.10).

### Longitudinal changes in HRQoL and mental health and associated risk and resource factors

A series of panel linear regression mixed models with random intercepts were conducted for each mental health outcome score. Table 2 shows the regression coefficients of the full models for HRQoL (*M* = 44.77; SD = 8.49), mental health problems (*M* = 9.93; SD = 6.26), anxiety (*M* = 5.95; SD = 4.42), depressive symptoms (*M* = 11.63; SD = 3.95) and psychosomatic complaints (*M* = 1.57; SD = 0.62). The corresponding results for the SDQ subscales can be found in Supplementary Table 2. The magnitude of the effects can be judged by comparing the regression coefficients with the actual standard deviation in the measures. According to Cohen [36], 0.2 SD can be classified as small, 0.5 SD as medium and 0.8 as large effect size. Time across wave 2 versus wave 1 was associated with statistically significant lower HRQoL (− 0.77 or − 0.09 SD), stronger emotional problems (+ 0.18 or + 0.08 SD) and peer problems (+ 0.10 or + 0.05 SD), more pronounced symptoms of anxiety (+ 0.45 or + 0.10 SD) and depression (+ 0.46 or + 0.12 SD), and stronger psychosomatic complaints (+ 0.10 or + 0.16 SD) such as irritability, headaches and sleeping problems. The effect thus was strongest for psychosomatic complaints (the coefficient indicates a significant increase of 0.10 points on the HBSC-SCL, which corresponds to an increase of 16% of a SD from wave 1 to wave 2). Contrary to a priori expectations, no statistically significant increase was found for mental health problems overall or for conduct problems; the level of hyperactivity problems even decreased from wave 1 to wave 2.

**Table 2:** Predictors of HRQoL and mental health in children and adolescents during the pandemic

	HRQoL^ac^			Mental health problems^bd^			Anxiety^ad^			Depressive symptoms^ad^			Psychosomatic complaints^ad^		
	B	95% CI		B	95% CI		B	95% CI		B	95% CI		B	95% CI	
		LL	UL		LL	UL		LL	UL		LL	UL		LL	UL
Intercept	46.35	45.54	47.16	9.14	8.75	9.52	5.10	4.63	5.55	10.53	10.14	10.91	1.36	1.30	1.42
Effect of time	− 0.77*	− 1.26	− 0.28	0.15	− 0.10	0.39	0.45*	0.20	0.70	0.46*	0.23	0.70	0.10*	0.07	0.14
Female	2.28	− 2.97	7.54	− 2.22*	− 3.90	− 0.54	− 2.68*	− 5.69	0.33	− 1.36	− 3.87	1.16	− 0.17	− 0.58	0.24
Age	0.22	− 0.03	0.48	− 0.48*	− 0.57	− 0.39	− 0.24*	− 0.39	− 0.09	− 0.05	− 0.18	0.07	− 0.01	− 0.03	0.01
Female × age	− 0.23	− 0.60	0.13	0.11	− 0.02	0.24	0.27*	0.07	0.48	0.14	− 0.03	0.32	0.02	− 0.01	0.05
Migration background	− 0.97	− 1.98	0.05	0.37	− 0.22	0.97	0.02	− 0.57	0.60	0.24	− 0.24	0.73	0.08*	0.01	0.16
Low parental education	0.71	− 0.22	1.64	1.07*	0.54	1.60	− 0.18	− 0.71	0.34	− 0.20	− 0.64	0.25	− 0.04	− 0.12	0.03
Single parenthood	− 1.19*	− 2.12	− 0.26	1.02*	0.44	1.59	0.67*	0.12	1.21	0.64*	0.20	1.09	0.12*	0.04	0.19
Living space	0.02	− 0.01	0.04	− 0.02*	− 0.03	− 0.01	− 0.02*	− 0.03	− 0.00	− 0.01	− 0.02	0.01	− 0.00	− 0.00	0.00
Parental mental illness	− 1.33*	− 2.64	− 0.02	2.00*	1.28	2.72	1.22*	0.50	1.94	1.18*	0.55	1.81	0.16*	0.06	0.26
Parental burden due to the pandemic	− 2.36*	− 3.56	− 1.16	1.76*	1.14	2.38	1.30*	0.66	1.94	1.29*	0.71	1.86	0.16*	0.07	0.25
Changes in occupational status	− 1.53*	− 2.34	− 0.72	0.75*	0.33	1.18	0.42	− 0.01	0.86	0.83*	0.44	1.22	0.11*	0.05	0.18
Family conflicts	− 2.24*	− 3.90	− 0.59	2.43*	1.64	3.23	0.57	− 0.32	1.45	1.48*	0.69	2.28	0.28*	0.16	0.41
Escalation of conflicts	− 1.20	− 2.56	0.16	1.40*	0.72	2.07	1.01*	0.29	1.72	0.81*	0.16	1.46	0.17*	0.06	0.27
Family climate^e^	3.53*	2.83	4.23	− 2.63*	− 3.01	− 2.25	− 0.98*	− 1.36	− 0.60	− 1.47*	− 1.81	− 1.14	− 0.21*	− 0.26	− 0.16
Social support^e^	2.85*	2.28	3.42	− 1.49*	− 1.81	− 1.18	− 0.59*	− 0.90	− 0.28	− 0.88*	− 1.15	− 0.60	− 0.09*	− 0.13	− 0.05
Model fit (adj. *R*^2^)	0.657			0.811			0.460			0.625			0.675		

The parameter estimates indicate how the predictors are associated with the outcomes (controlling for the other predictors)

^a^Self-report, 11–17 years

^b^Parent-report, 7–17 years

^C^Higher values indicate better HRQoL

^d^Higher values indicate stronger symptoms and complaints

^e^Higher values indicate stronger resources; * *p* < 0.05

The full model included a set of additional potential determinants and thus statistically controls for confounding factors: female gender and older age were associated with fewer mental health problems, fewer emotional problems, less hyperactivity, fewer conduct problems and fewer symptoms of anxiety during the pandemic. The effect of female gender was strongest for symptoms of anxiety (minus 61% of a SD on the SCARED). However, a significant interaction between gender and age indicated that older females reported more symptoms of anxiety. No gender or age effects were found for HRQoL, depressive symptoms or psychosomatic complaints.

In terms of risk factors, migration background was connected with stronger and psychosomatic complaints in children and adolescents (plus 13% of a SD on the HBSC-SCL). Low parental education was associated with stronger mental health problems (plus 17% of a SD on the SDQ). Single parenthood was associated with lower HRQoL (− 0.14 SD), increased mental health problems (+ 0.16 SD), increased symptoms of anxiety (+ 0.15 SD) and depression (+ 0.16 SD) and more frequent psychosomatic complaints, with the effect being strongest for psychosomatic complaints (plus 19% of a SD on the HBSC-SCL). Larger living space was associated with fewer mental health problems and symptoms of anxiety (every 10 square meters more were associated with -0.03 SD and − 0.05 SD). Parental mental illness was related to lower HRQoL (− 0.16 SD) and more pronounced mental health problems (+ 0.32 SD), anxiety (+ 0.28 SD), depressive symptoms (+ 0.30 SD) and psychosomatic complaints (+ 0.26 SD).

Concerning pandemic-related risk factors, parental burden caused by the pandemic and by changes in occupational status, family conflicts and the escalation of conflicts were all related to impairments in almost all HRQoL and mental health outcomes. The strongest effects were found for the risk factor family conflicts on psychosomatic complaints (plus 45% of a SD on the HBSC-SCL) and on depressive symptoms (plus 37% of a SD on the CES-DC).

With regard to resource factors, a more positive family climate and higher levels of social support were each associated with higher HRQoL (+ 0.42 SD and + 0.34 SD) and less pronounced mental health problems (− 0.42 SD and -0.24 SD), anxiety (− 0.22 SD and − 0.13 SD), depressive symptoms (− 0.37 SD and − 0.22 SD) and psychosomatic complaints (− 0.34 SD and − 0.14 SD). The effects of family climate and social support were strongest for HRQoL and mental health problems.

The inclusion of the interaction terms did not provide a clear indication of whether the time-constant factors moderated longitudinal changes in HRQoL and mental health outcomes (see Supplementary Table 3). Higher age was associated with a decline in HRQoL but also with fewer peer problems from wave 1 to wave 2. Parental mental illness was related to significantly increased peer problems in children and adolescents across the waves. Migration background explained a significant increase in the symptoms of anxiety from wave 1 to wave 2, while a larger living space was associated with fewer psychosomatic complaints.

In subsequent panel model analyses, the role of certain aspects of social distancing measures was examined for our outcomes. The analyses were controlled for age, gender, age*gender interaction and time (wave 2 vs. wave 1). Compared to children attending school most of the time, children who were in school part-time or infrequently for most of the time showed increasing mental health problems (overall: + 1.04 or 0.15 SD, emotional problems: + 0.29 or 0.13 SD, hyperactivity: + 0.34 or 0.14 SD, peer problems: + 0.22 or 0.12 SD). Slightly reduced social contacts were related to impaired HRQoL (− 2.86 or 0.34 SD), whereas much reduced social contacts were associated with a further decrease in HRQoL (− 5.09 or 0.60 SD), increasing mental health problems overall (+ 0.70 or 0.11 SD), emotional problems (+ 0.21 or 0.09 SD) and hyperactivity (+ 0.34 or 0.14 SD), as well as increasing symptoms of anxiety (+ 0.92 or 0.21 SD), depressive symptoms (+ 0.74 or 0.19 SD) and psychosomatic complaints (+ 0.12 or 0.20 SD). There was a gradient towards lower HRQoL and stronger mental health problems regarding how often the children and adolescents were outside on a weekly basis. Compared with children being outside almost every day, children who were outside 0 days a week had lower HRQoL (− 4.30 or − 0.51 SD), increasing mental health problems (overall: + 2.18 or 0.35 SD, emotional problems: + 0.68 or 0.31 SD, conduct problems: + 0.49 or 0.18 SD, hyperactivity: + 0.43 or 0.18 SD, peer problems: + 0.76 or 0.41 SD) as well as increased symptoms of anxiety (+ 0.98 or 0.22 SD) symptoms of depression (+ 1.96 or 0.50 SD) and psychosomatic complaints (+ 0.22 or 0.35 SD).

## Discussion

The German COPSY study is one of the first population-based longitudinal studies on the HRQoL and mental health of children and adolescents during the COVID-19 pandemic. In the first wave, we found that children and adolescents experienced lower HRQoL and significantly more mental and psychosomatic health problems than before the pandemic [4]. In the second wave, the HRQoL of these children and adolescents decreased further, and emotional problems, peer problems, anxiety, depressive symptoms and psychosomatic complaints increased significantly. There were larger changes from pre-pandemic to pandemic data while the changes from wave 1 to wave 2 are rather modest, but still indicated that the observed impairments in well-being and mental health seem to be rather stable over the course of the pandemic. The findings also indicate that children missing school frequently or having less social contacts due to lockdown measures as well as socially disadvantaged children and children of mentally ill parents are at risk of being particularly burdened. Furthermore, a positive family climate and social support were each associated with better HRQoL and mental health during the pandemic.

The findings can be interpreted as a negative mental health impact of the pandemic, but a true causal relation is not proven. It cannot be ruled out that pandemic-related factors (like regional differences in infection rates or lockdown measures) or factors other than the pandemic (like seasonal changes, growing older during the study, etc.) could also have led to the higher rates of mental health problems found in the present study. However, our results are roughly in line with systematic reviews of cross-sectional studies on the impact of the pandemic [2, 3]. Those reviews have reported an increase of mental health problems, with only very few studies reporting no negative mental health impact during the pandemic. Thus, it is likely that our study describes a negative mental health effect, which is related to the pandemic. Comparing our results to the prevalences found in the reviews of cross-sectional studies, we find that in our study the anxiety levels were higher (30%) and the depression levels were lower (15%) than the pooled average of the reviews [2, 3]. Further research is needed to explore what may have led to those differences.

Comparing our study to the very few longitudinal studies, the Co-SPACE study [10] found an increase in conduct problems and stronger hyperactivity problems, but a slightly lower increase in emotional problems during the pandemic than in the COPSY study. The difference in the increase in mental health problems may be due to differences in measurement points, infection rates, lockdown measures and access to health care and financial support between the countries. The longitudinal NY study on adolescents [11] found an initial increase in anxiety and depression a few weeks after the peak of infection rates, followed by a decrease of anxiety and depression after home confinement and school closures were in place and infection rates went down. Hawes et al. [11] cautiously discuss that the impact of infection rates may occur through downstream mechanisms that trail infection rates (e.g., policy changes, spread of information). Our study also found an initial increase of anxiety and depression levels, similar to those of Hawes et al. [11]. However, as we did not have official infection rates, but only the knowledge of the pandemic waves in Germany, we cannot discuss our findings in relation to those rates in detail, but call for future studies to explore the link between infection rates and mental health problems in children more closely. In line with Hawes et al. [11] who found that individuals who reported more school and home confinement concerns had more severe depressive and anxiety symptoms, our study revealed that children and adolescents who attended school less frequently due to lockdown measures or stayed at home more often reported more mental health problems. Moreover, the most recent longitudinal Icelandic study [37] found an increase in depressive symptoms and a deterioration in mental well-being in 10/2020 during the pandemic compared to pre-pandemic data—similar to our and Hawes’ [11] initial finding of worsening mental health.

Although the pandemic (among potential other factors) appears to increase overall mental distress in children and adolescents, no changes in the age- and gender-specific distribution of mental health problems was found during the pandemic. In line with the results of population-based studies conducted before the pandemic [38], externalizing problems such as hyperactivity were more pronounced in boys than girls and in younger children than older children, while internalizing problems such as anxiety were more likely in girls than boys and in older children than younger children. Thus, the age- and gender-sensitive diagnosis and treatment of mental health problems is very important in clinical practice, even in times of pandemic.

The COPSY study also showed that both children from socially disadvantaged families and children of parents with mental health problems had a significantly lower HRQoL and more pronounced mental health problems during the pandemic. We further found that pandemic-related changes in parental occupational status and family conflicts were associated with impaired HRQoL and mental health in children and adolescents. This is in line with the results of the German COVID-19 Snapshot Monitoring (COSMO) [39], which found that the pandemic poses major challenges for many families.

Our study results emphasize that children who are at risk need to be identified and supported at an early stage to avoid the progression of mental health problems to mental disorders. We know from a large body of research that social inequality is related to mental health [40]. Recent studies also indicate that there are social inequalities in the risk of infection with COVID-19 [41]. To reduce the identified health inequalities, we suggest that targeted and low-threshold prevention and early intervention measures should be initiated for children from socially deprived backgrounds. Special programs are needed that include financial support, additional childcare, health care and outdoor activity outreach programs. Furthermore, educational support needs to be expanded and services such as virtual parent consultation hours, tutoring services and specialized and individualized support for students with special educational needs should be implemented. Schools, daycares, parents, doctors, therapists, social workers, sports coaches and society as a whole can attempt to stay in contact with and support children at risk. Furthermore, as the mental health of children and their parents is closely intertwined [42], parents need to be encouraged to seek help and use counseling and crisis services. In this context, the thresholds for accessing support services need to be lowered, and mental health support needs to be destigmatized.

While the COPSY study shows that the majority of children are burdened during the pandemic and that a considerable number of children are at risk, we also identified a positive family climate and social support as important resources that strengthen their HRQoL and mental health during the pandemic. Children in cohesive families who spend time together and children who feel supported by their social environment are better able to cope with the challenges of the crisis. It seems that psychosocial resources and resilience factors, which have already proven their protective effect in previous studies [43], are also able to protect children’s mental health in times of crisis.

There are a number of strengths and limitations related to our study. The strengths of the study include the large population-based sample; the assessment of HRQoL and mental health using established and validated instruments; and the availability of population-based pre-pandemic reference samples. The limitations include the fact that data were assessed during two short periods at wave 1 (late spring) and wave 2 (winter), thus seasonal effects may be possible. The pandemic burden was assessed with newly, not yet psychometrically validated items and mental health symptoms were measured using screening questionnaires; thus no clinical diagnoses were assessed. The COPSY study identified associations and not cause–effect relationships. Also, multiple analyses may have increased the likelihood of significant findings. Finally, our results may not be generalizable to countries other than Germany.

Overall, the findings of the COPSY study highlight the increasing mental health burden on children and adolescents during the COVID-19 pandemic. Although it can be assumed that not all children with mental health problems will develop a mental disorder, the results are highly relevant to public health and policy. As our society has not thus far succeeded in stabilizing or improving the negative mental health burden during the pandemic of children and adolescents, we strongly recommend carefully balancing lockdown and social distancing measures with children’s mental health risks. By that we mean to take the well-being and mental health of children more into account and not to lower infection prevention measures per se as they prevent high incidences and consequently deaths. We also call for the development of targeted and low-threshold health promotion, prevention and early intervention programs to support children and adolescents who have been severely affected by the pandemic. This is a task that needs to be addressed by the entire society, including politicians and education and health care professionals, to protect, restore, and maintain the mental health of children and adolescents.

## Supplementary Information

Below is the link to the electronic supplementary material.

Supplementary file1 (PDF 341 KB)

## Data Availability

The data that support the findings of this study are available from the corresponding author upon reasonable request.

## Declarations

### Exclusion of data

This paper reports all outcomes pertaining to mental health and subjective well-being that have been measured in the COPSY study. Regarding the predictors, we did not include every piece of information gathered from the respondents (items such as physical activity and media use) but included those which—from a conceptual point of view—could be considered potential determinants, confounders or important contextual factors. The results of items asking about health behaviors (such as physical activity and media use) will be part of a later publication.
