# Supplementary material for: Quality of life and mental health in children and adolescents during the first year of the COVID-19 pandemic: results of a two-wave nationwide population-based study
Source: Eur Child Adolesc Psychiatry. 2021 Oct 12;32(4):575–88. doi: 10.1007/s00787-021-01889-1 (PMC8506100; doi:10.1007/s00787-021-01889-1)
Supplement: Supplementary file 1 — Supplementary file1 (PDF 341 KB) [file 787_2021_1889_MOESM1_ESM.pdf]

Supplementary Material

Manuscript title: Quality of life and mental health in children and adolescents during the first year of the COVID-19 pandemic: Results of a two-wave nationwide population-based study

Journal: European Child and Adolescent Psychiatry

Authors: Ulrike Ravens-Sieberer, Anne Kaman, Michael Erhart, Christiane Otto, Janine Devine, Constanze Löffler, Klaus Hurrelmann, Monika Bullinger, Claus Barkmann, Nico Siegel, Anja Simon, Lothar H. Wieler, Robert Schlack, Heike Hölling

Corresponding author: Prof. Dr. Ulrike Ravens-Sieberer (ravens-sieberer@uke.de)

Supplementary Table 1. Self-developed COVID-19 pandemic-specific items

Construct	Item	Response categories
Overall burden (children)	How difficult / burdensome were the changes related to the pandemic for you overall?	1 (“not at all difficult / burdensome”) to 5 (“very difficult / burdensome”)
Overall burden (parents)	How burdensome were the changes related to the pandemic for you overall?	1 (“not at all burdensome”) to 5 (“very burdensome”)
School closures (children)	How do you perceive schooling and learning / work now compared to a regular school or work day?	1 (“a lot more difficult“) to 5 (“much less difficult“)
Social contacts (children)	How much are you in contact with your friends or people outside your family compared to before the pandemic?	1 (“much less”) to 5 (“a lot more)
	How difficult / burdensome were those changes in relationships to your friends for you?	1 (“not difficult / burdensome at all” to 5 (“very difficult / burdensome”)
Family conflicts (children)	To what degree did the frequency of arguments in your family change compared to before the pandemic?	1 (“a lot more”) to 5 (“much less”)
	Sometimes on special occasions arguments escalate unfortunately between parents and children. Compared to before the pandemic this happened to us…	1 (“much less”) to 5 (“a lot more”) and 6 (“this does not happen at all”)
Changes in occupational status (parents)	How burdensome were the changes in your occupational status for you?	1 (“not at all burdensome”) to 5 (“very burdensome”)

Supplementary Table 2. Predictors of emotional problems, conduct problems, hyperactivity and peer problems in children and adolescents during the pandemic

	Emotional problems[ab]			Conduct problems[ab]			Hyperactivity[ab]			Peer problems[ab]		
	B	95 % CI		B	95 % CI		B	95 % CI		B	95 % CI	
		LL	UL		LL	UL		LL	UL		LL	UL
Intercept	*1.30*	*1.16*	*1.45*	*1.92*	*1.81*	*2.03*	*3.75*	*3.60*	*3.91*	*2.13*	*2.00*	*2.26*
Effect of time	0.18*	0.09	0.28	-0.01	-0.09	0.07	-0.12*	-0.22	-0.03	0.10*	0.01	0.18
Female	-0.63*	-1.26	-0.01	-0.56*	-1.04	-0.08	-0.83*	-1.50	-0.17	-0.18	-0.75	0.39
Age	-0.13*	-0.16	-0.10	-0.11*	-0.14	-0.08	-0.24*	-0.28	-0.21	0.01	-0.03	0.04
Female*age	0.08*	0.03	0.13	0.03	-0.01	0.07	0.01	-0.04	0.06	-0.01	-0.05	0.04
Migration background	0.13	-0.10	0.35	0.12	-0.05	0.29	0.01	-0.23	0.24	0.11	-0.09	0.32
Low parental education	0.28*	0.08	0.48	0.26*	0.11	0.42	0.35*	0.14	0.56	0.21*	0.03	0.39
Single parenthood	0.25*	0.04	0.47	0.17*	0.01	0.33	0.24*	0.01	0.47	0.34*	0.15	0.54
Living space	-0.01*	-0.01	0.00	0.00	-0.01	0.00	-0.01	-0.01	0.00	0.00*	-0.01	0.00
Parental mental illness	0.87*	0.59	1.15	0.30*	0.08	0.52	0.35*	0.06	0.63	0.55*	0.30	0.80
Parental burden due to the pandemic	0.69*	0.44	0.93	0.27*	0.07	0.46	0.53*	0.29	0.78	0.31*	0.10	0.53
Changes in occupational status	0.31*	0.14	0.47	0.19*	0.06	0.32	0.18*	0.02	0.35	0.10	-0.04	0.25
Family conflicts	0.89*	0.57	1.21	0.75*	0.51	1.00	0.68*	0.37	0.99	0.23	-0.05	0.50
Escalation of conflicts	0.48*	0.21	0.74	0.64*	0.43	0.85	0.27*	0.01	0.54	0.09	-0.14	0.32
Family climate[c]	-0.65*	-0.80	-0.50	-0.79*	-0.90	-0.67	-0.85*	-0.97	-0.68	-0.42*	-0.55	-0.29
Social support[c]	-0.37*	-0.49	-0.24	-0.32*	-0.41	-0.22	-0.39*	-0.52	-0.27	-0.43*	-0.54	-0.33
Model fit (adj. R^2)	0.699			0.691			0.796			0.708		

Note. [a] parent-report, 7-17 years; [b] higher values indicate stronger symptoms; [c] higher values indicate stronger resources; * $p < .05$

Supplementary Table 3. Time-constant factors moderating the relationships between wave 2 and HRQoL and mental health in children and adolescents during the pandemic

	HRQoL[ac]			Mental health problems[bd]			Anxiety[ad]			Depressive symptoms[ad]			Psychosomatic complaints[ad]			Emotional problems[bd]			Conduct problems[bd]			Hyperactivity[bd]			Peer problems[bd]		
	B	95 % CI		B	95 % CI		B	95 % CI		B	95 % CI		B	95 % CI		B	95 % CI		B	95 % CI		B	95 % CI		B	95 % CI	
		LL	UL		LL	UL		LL	UL		LL	UL		LL	UL		LL	UL		LL	UL		LL	UL		LL	UL
Intercept	*45.97*	*45.05*	*46.88*	*9.21*	*8.79*	*9.62*	*5.34*	*4.83*	*5.85*	*10.74*	*10.30*	*11.18*	*1.39*	*1.32*	*1.46*	*1.33*	*1.17*	*1.49*	*1.96*	*1.84*	*2.08*	*3.75*	*3.58*	*3.91*	*2.14*	*1.99*	*2.28*
Effect of time	-0.13	-1.08	0.82	0.03	-0.36	0.41	0.04	-0.44	0.53	0.08	-0.38	0.54	0.05	-0.02	0.12	0.14	-0.02	0.29	-0.09	-0.21	0.04	-0.11	-0.26	0.04	0.09	-0.05	0.22
Female	2.58*	-2.69	7.84	-2.23*	-3.92	-0.55	-2.80	-5.83	0.22	-1.48	-4.00	1.03	-0.19	-0.60	0.22	-0.64*	-1.27	-0.01	-0.56*	-1.04	-0.08	-0.85*	-1.51	-0.18	-0.18	-0.75	0.39
Age	0.42*	0.12	0.72	-0.47*	-0.57	-0.37	-0.32*	-0.49	-0.15	-0.12	-0.27	0.02	-0.02	-0.04	0.01	-0.14*	-0.18	-0.10	-0.11*	-0.13	-0.08	-0.25*	-0.29	-0.21	0.02	-0.01	0.06
Female*age	-0.24	-0.60	0.13	0.11	-0.02	0.25	0.29*	0.08	0.50	0.14	-0.03	0.32	0.02	-0.01	0.05	0.08*	0.03	0.13	0.03	-0.01	0.06	0.01	-0.04	0.06	-0.01	-0.05	0.04
Migration background	-1.10	-2.33	0.13	0.18	-0.51	0.86	-0.50	-1.18	0.19	0.23	-0.36	0.82	0.08	-0.02	0.17	0.07	-0.19	0.34	0.02	-0.18	0.22	0.02	-0.25	0.29	0.05	-0.18	0.29
Low parental education	0.08	-1.05	1.21	1.06*	0.44	1.68	-0.33	-0.95	0.29	-0.08	-0.62	0.46	-0.04	-0.13	0.04	0.28*	0.04	0.52	0.17	-0.02	0.36	0.46*	0.22	0.71	0.19	-0.03	0.40
Single parenthood	-0.84	-1.95	0.27	0.95*	0.30	1.60	0.66*	0.04	1.28	0.38	-0.15	0.91	0.06	-0.02	0.15	0.17	-0.08	0.41	0.23*	0.03	0.42	0.21	-0.05	0.46	0.34*	0.12	0.56
Living space	0.01	-0.02	0.04	-0.03*	-0.04	-0.01	-0.01	-0.03	0.00	0.00	-0.01	0.02	0.00*	0.00	0.00	-0.01	-0.02	0.00	-0.01	-0.01	0.00	0.00	-0.01	0.00	-0.01	-0.01	0.00
Parental mental illness	-1.92*	-3.60	-0.23	1.83*	0.93	2.73	1.01*	0.11	1.91	1.22*	0.41	2.03	0.20*	0.07	0.33	0.83*	0.48	1.18	0.37*	0.09	0.64	0.37*	0.02	0.73	0.33*	0.03	0.64
Parental burden due to the pandemic	-2.36*	-3.56	-1.16	1.76*	1.14	2.38	1.27*	0.63	1.91	1.28*	0.70	1.86	0.16*	0.07	0.25	0.69*	0.45	0.94	0.26*	0.07	0.45	0.53*	0.28	0.77	0.32*	0.11	0.53
Changes in occupational status	-1.50*	-2.30	-0.69	0.75*	0.33	1.17	0.43*	0.00	0.87	0.82*	0.44	1.21	0.11*	0.05	0.18	0.30*	0.14	0.47	0.19*	0.06	0.31	0.18*	0.02	0.35	0.11	-0.04	0.25
Family conflicts	-2.12*	-3.78	-0.46	2.40*	1.60	3.20	0.52	-0.37	1.40	1.45*	0.65	2.24	0.28*	0.16	0.40	0.86*	0.54	1.18	0.76*	0.51	1.00	0.67*	0.36	0.99	0.23	-0.05	0.51
Escalation of conflicts	-1.32	-2.69	0.04	1.39*	0.72	2.06	0.98*	0.26	1.69	0.84*	0.19	1.49	0.17*	0.07	0.27	0.48*	0.21	0.75	0.64*	0.43	0.84	0.27*	0.01	0.54	0.08	-0.15	0.32
Family climate[e]	3.49*	2.79	4.19	-2.64*	-3.02	-2.26	-0.97*	-1.35	-0.60	-1.46*	-1.79	-1.12	-0.21*	-0.26	-0.16	-0.65*	-0.80	-0.50	-0.79*	-0.91	-0.68	-0.82*	-0.97	-0.67	-0.43*	-0.56	-0.30
Social support[e]	2.85*	2.28	3.42	-1.49*	-1.80	-1.18	-0.61*	-0.92	-0.30	-0.89*	-1.16	-0.61	-0.09*	-0.14	-0.05	-0.37*	-0.49	-0.24	-0.32*	-0.41	-0.22	-0.40*	-0.52	-0.27	-0.43*	-0.54	-0.32
Wave 2*Female	-0.39	-1.37	0.59	0.02	-0.46	0.51	-0.11	-0.61	0.39	0.20	-0.27	0.68	0.03	-0.04	0.11	-0.02	-0.22	0.18	0.09	-0.07	0.25	0.04	-0.15	0.23	-0.09	-0.26	0.08
Wave 2*Age	-0.33*	-0.58	-0.07	-0.01	-0.08	0.06	0.12	-0.01	0.25	0.11	-0.01	0.24	0.01	-0.01	0.03	0.02	-0.01	0.05	-0.01	-0.03	0.02	0.02	-0.01	0.05	-0.04*	-0.06	-0.01
Wave 2*Migration background	0.32	-1.07	1.71	0.37	-0.31	1.05	1.01*	0.31	1.72	0.02	-0.65	0.69	0.02	-0.08	0.12	0.09	-0.18	0.37	0.18	-0.04	0.40	-0.02	-0.29	0.24	0.12	-0.12	0.35
Wave 2*Low parental education	1.26	-0.04	2.56	0.00	-0.65	0.65	0.30	-0.36	0.96	-0.21	-0.84	0.42	0.00	-0.09	0.10	0.00	-0.26	0.27	0.18	-0.03	0.39	-0.21	-0.47	0.04	0.02	-0.20	0.25
Wave 2*Single parenthood	-0.73	-1.96	0.50	0.15	-0.48	0.78	0.00	-0.62	0.62	0.53	-0.06	1.13	0.11*	0.02	0.20	0.19	-0.07	0.44	-0.12	-0.32	0.09	0.07	-0.18	0.32	0.01	-0.21	0.24
Wave 2*Living space	0.01	-0.03	0.04	0.01*	0.00	0.03	0.00	-0.02	0.01	-0.01	-0.03	0.00	0.00	-0.01	0.00	0.01*	0.00	0.01	0.01*	0.00	0.01	0.00	-0.01	0.01	0.00*	0.00	0.01
Wave 2*Parental mental illness	1.07	-1.02	3.16	0.34	-0.68	1.36	0.42	-0.65	1.49	-0.03	-1.04	0.97	-0.06	-0.22	0.09	0.09	-0.33	0.50	-0.14	-0.47	0.19	-0.03	-0.44	0.37	0.41*	0.05	0.76
Model fit (adj. R^2)	0.663			0.812			0.464			0.628			0.682			0.701			0.692			0.796			0.712		

Note. [a] self-report, 11-17 years; [b] parent-report, 7-17 years; [c] higher values indicate better HRQoL; [d] higher values indicate stronger symptoms and complaints; [e] higher values indicate stronger resources; * $p < .05$
